# Baseline Predictors of Mortality among Predominantly Rural-Dwelling End-Stage Renal Disease Patients on Chronic Dialysis Therapies in Limpopo, South Africa

**DOI:** 10.1371/journal.pone.0156642

**Published:** 2016-06-14

**Authors:** Ramon A. Tamayo Isla, Oluwatoyin I. Ameh, Darlington Mapiye, Charles R. Swanepoel, Aminu K. Bello, Andrew R. Ratsela, Ikechi G. Okpechi

**Affiliations:** 1 Polokwane Kidney and Dialysis Centre, Pietersburg Provincial Hospital and the University of Limpopo, Polokwane, South Africa; 2 Division of Nephrology and Hypertension, University of Cape Town, Cape Town, South Africa; 3 South African National Bioinformatics Institute/Medical Research Council of South Africa Bioinformatics Unit, University of the Western Cape, Cape Town, South Africa; 4 Division of Nephrology, Department of Medicine, University of Alberta, Edmonton, Canada; Postgraduate Medical Institute, INDIA

## Abstract

**Background:**

Dialysis therapy for end-stage renal disease (ESRD) continues to be the readily available renal replacement option in developing countries. While the impact of rural/remote dwelling on mortality among dialysis patients in developed countries is known, it remains to be defined in sub-Saharan Africa.

**Methods:**

A single-center database of end-stage renal disease patients on chronic dialysis therapies treated between 2007 and 2014 at the Polokwane Kidney and Dialysis Centre (PKDC) of the Pietersburg Provincial Hospital, Limpopo South Africa, was retrospectively reviewed. All-cause, cardiovascular, and infection-related mortalities were assessed and associated baseline predictors determined.

**Results:**

Of the 340 patients reviewed, 52.1% were male, 92.9% were black Africans, 1.8% were positive for the human immunodeficiency virus (HIV), and 87.5% were rural dwellers. The average distance travelled to the dialysis centre was 112.3 ± 73.4 Km while 67.6% of patients lived in formal housing. Estimated glomerular filtration rate (eGFR) at dialysis initiation was 7.1 ± 3.7 mls/min while hemodialysis (HD) was the predominant modality offered (57.1%). Ninety-two (92) deaths were recorded over the duration of follow-up with the majority (34.8%) of deaths arising from infection-related causes. Continuous ambulatory peritoneal dialysis (CAPD) was a significant predictor of all-cause mortality (HR: 1.62, CI: 1.07–2.46) and infection-related mortality (HR: 2.27, CI: 1.13–4.60). On multivariable cox regression, CAPD remained a significant predictor of all-cause mortality (HR: 2.00, CI: 1.29–3.10) while the risk of death among CAPD patients was also significantly modified by diabetes mellitus (DM) status (HR: 4.99, CI: 2.13–11.71).

**Conclusion:**

CAPD among predominantly rural dwelling patients in the Limpopo province of South Africa is associated with an increased risk of death from all-causes and infection-related causes.

## Introduction

Renal transplantation remains the most cost-effective treatment option in end-stage renal disease (ESRD), providing a better quality of life and extending survival. It however still remains a poorly utilized option in developing countries due to issues of poor infrastructure, technical manpower shortages, and cultural and religious beliefs about organ donation.[1, 2] Chronic dialysis therapies (hemodialysis and peritoneal dialysis) therefore remain the more readily available treatment options for ESRD patients in developing countries. Among countries in sub-Saharan Africa (SSA) in which maintenance dialysis is available, haemodialysis (HD), rather than peritoneal dialysis (PD), is often the modality of choice, mainly due to the high cost of performing and maintaining continuous ambulatory peritoneal dialysis (CAPD) programmes, unavailability of peritoneal dialysis fluids in some countries, an increased risk of peritonitis given the generally high burden of infectious diseases, and lower physician reimbursement for CAPD.[3–5]

Dialysis patients have a higher mortality risk than matched individuals in the general population with the identified factors for the increased risk including race, dialysis modality and the inordinate occurrence of both traditional (hypertension, diabetes, dyslipidemia) and non-traditional (anemia, fluid overload, calcium-phosphate imbalance) cardiovascular (CV) risk factors among these patients.[6, 7] Higher mortality rates have been observed among Caucasian patients while PD appears to confer a survival advantage relative to HD, especially among non-diabetics—an advantage which declines with increasing dialysis vintage.[8, 9] Survival outcomes among maintenance dialysis patients in developing countries have shown a greater annual cumulative survival among HD patients in comparison to CAPD patients (73.4% versus 62%).[10] Data from South Africa however shows similar cumulative survival (approximately 88%) for both modalities.[10] Infection in addition to CV factors are the leading identified causes of death.

Access to renal replacement modalities is a challenge in developing countries due to scarce resources and competing economic choices which limit the number of dialysis centers within communities/regions. In addition to determining the associations between patient-related and modality-related factors and mortality, focus has also centered on the relationship between proximity to health care services and hard patient outcomes such as mortality in CKD and ESRD. Thompson et al demonstrated that chronic dialysis patients living greater than 100 miles from dialysis centres in the United States of America had a 21.0% increased risk of mortality; no association was however shown between dwelling (rural or urban) and dialysis-related mortality.[11]

The determinants of mortality among rural dwelling South Africans on maintenance dialysis therapies is unknown and we thus sought to identify the prevailing causes and predictors of mortality among predominantly rural-dwelling ESRD patients in the Limpopo province of South Africa.

## Methods

### Study population

This study is a retrospective analysis of a single-center ESRD database of patients treated with HD or CAPD at the Polokwane Kidney and Dialysis Centre (PKDC) of the Pietersburg Provincial Hospital, Limpopo South Africa. The study received approval from the Pietersburg Provincial Hospital Ethics Committee.

The period under review was from 2007 (when PKDC was commissioned) to July 2014. All incident dialysis patients treated at the unit during the period under review were included. Patients were included if they had been on stable dialysis for at least 3 months. Twenty (20) patients who had started dialysis before 2007 at other centres and who continued dialysis with the unit due to proximity considerations were also included in our review. The outcomes of 340 patients were thus determined. Dialysis modality at day 91 after dialysis initiation (60 days of which the patient would have continuously been on the modality) was taken as the predominant modality. Duration of follow up for each modality was calculated from the date of starting dialysis to date of last date of follow up (31^st^ July 2014), or date of transplantation, or date of death.

### Covariates

We collected relevant socio-demographic data such as age at initiation of dialysis, gender, marital status, race, socio-economic status, predominant area of dwelling (urban versus rural), and housing characteristics (formal or informal). Baseline (at dialysis initiation) clinical and biochemical parameters collected included weight, body mass index (BMI), systolic and diastolic blood pressures, estimated glomerular filtration rate (eGFR), serum albumin, serum cholesterol, serum ferritin, transferrin saturation, calcium, inorganic phosphate, and parathyroid hormone. The cause of ESRD was also ascertained. The cause was reported as unknown in those patients who were late presenters; however if the cause despite late presentation was apparent (e.g. polycystic kidney disease) a definite cause was allotted.

### Outcomes and Definitions

Death within each modality was the outcome of interest of this study. Causes of death were divided into CV, infection-related or other causes. We defined CV death as death resulting from stroke, heart failure, myocardial infarction, pulmonary embolism or any death reported to have occurred suddenly with no other known cause. Infection-related deaths were deaths related to any ongoing infection at the time of death and these included peritonitis (for patients on PD), pneumonia, tuberculosis, catheter-related blood stream infection(CRBSI), infective endocarditis (one case) and empyema thoracis (one case). Deaths recorded as others were deaths at home that were not medically certified, and malignancy-related deaths.

### Statistical analysis

Patients’ data were anonymized and de-identified prior to statistical analysis. The data was analyzed using the Stata^®^ 13 software (Stata Corp, Texas). Continuous variables are presented as means and standard deviation (SD), and median with interquartile ranges where applicable. Categorical variables were presented as frequencies and percentages. Univariate analysis was performed using the independent student’s *t*-test, or Wilcoxon rank sum test where appropriate. Chi-squared test was used to compare categorical variables as appropriate. Kaplan-Meier survival analysis was used to determine the median time-to-survival of patients with the log rank test applied to explore significant difference in survival between dialysis modalities. Censoring occurred at loss-to-follow up, elective withdrawal from the dialysis program or kidney transplantation, whichever occurred first. Univariate Cox proportional regression analysis was performed to determine the association between baseline characteristics and all-cause and cause-specific mortalities (CV and infection-related mortalities). Variables with p<0.25 on univariate cox regression analysis were entered into the multivariable Cox regression to determine the baseline predictor(s) of all-cause mortality. Survival analysis was based on an intention-to-treat analysis hence modality switches during the study period were not taken into account. In assessing cause-specific mortalities, a cumulative incidence competing-risk analysis was utilized.[12] Missing data points on assessed covariates were adjudged to be missing at random and were subsequently multiply imputed. A *p*-value < 0.05 was considered statistical significant.

## Results

Table 1 summarises the baseline features of all the patients in this study. The average age was 36.1±11.9 years, with a slight preponderance of male patients (52.1%). The majority of patients (92.9%) were of black African ancestry while, in keeping with the geo-demography of Limpopo, many of the patients (87.5%) were rural dwellers. The average distance travelled from patients’ homes to the dialysis centre was 112.3 ± 73.4km. Approximately 60.0% of the patients were treated with HD (Table 1). As a result of late presentation, the cause of ESRD remained unknown in 45.0% while hypertension, diabetes mellitus and glomerulonephritis accounted for 25.9%, 10.3% and 6.8% respectively of all dialyzed patients. There was no difference in eGFR at dialysis initiation between HD and CAPD patients. Six (3.1%) of the patients on HD were positive for the human immunodeficiency virus (HIV) while there were no HIV positive patients on CAPD. Only 4 patients (1.1%) received kidney transplants (living donors) during the period of follow up. Other clinical characteristics of the patients are shown in Table 1.

**Table 1:** Baseline demographic and clinical characteristics of patients according to modality.

Baseline Characteristic	All Patients (n = 340)	HD patients (n = 194)	CAPD patient (n = 146)	p-value
Age at start of dialysis (Years)	36.1 ± 11.9	43.3 ± 12.0	35.3 ± 11.5	0.35
Gender (Male / Female) (%)	52.1/47.9	47.4/52.6	51.4/48.6	0.83
Race: (%)				
- Blacks	92.9	93.8	91.8	0.32
- Whites	5.0	4.6	5.5	
- Others**	2.1	1.6	2.7	
Predominant area of dwelling [rural locale] (%)	87.5	85.2	90.7	0.13
Type of housing [Formal] %	67.6	67.6	80.7	0.01*
Distance to Dialysis unit (km)	112.3 ± 73.4	110.7 ± 75.9	114.3 ± 70.2	0.66
BMI (kg/m^2^)	23.9 ± 5.5	23.6 ± 5.0	24.3 ± 6.3	0.37
eGFR [MDRD] (mls/min)	7.1 ± 3.7	7.0 ± 0.1	7.3 ± 0.1	0.11
SBP (mmHg)	140.1 ± 27.1	141.5 ± 26.3	137.9 ± 28.1	0.25
DBP (mmHg)	84.6 ± 17.7	84.4 ± 16.8	84.8 ± 19.0	0.87
Cause of ESRD: (%)				0.12
- Diabetes	10.3	11.3	8.9	
- Hypertension	25.9	22.7	30.1	
- Glomerulonephritis	6.8	8.3	4.8	
- Unknown	45.0	45.4	44.5	
- Others***	12.0	12.3	11.7	
Duration of follow up	36.6 ± 25.4	43.3 ± 26.3	27 ± 21.4	<0.001*^#^

* P < 0.05

**Others—Mixed ancestry, Indians and Asians

***Others—Unknown causes, Autosomal dominant polycystic kidney disease, Obstructive uropathy, chronic interstitial Nephritis

^#^ Wilcoxon rank sum test

Differences between HD and CAPD patients with respect to baseline socio-demographic and clinical parameters are also shown in Table 1. There was no difference in age, gender, race, or predominant area of dwelling between both groups. There was a statistically significant difference however in the type of housing, with more CAPD patients dwelling in formal houses (HD vs PD: 67.6% vs 80.7%, p = 0.01). Although the distance travelled to get to the dialysis unit was longer in the CAPD patients, this was not significantly different from the distance travelled by HD patients. The duration of follow-up was statistically shorter among PD patients (HD vs PD, 43.3 ± 26.3 months vs. 27.0 ± 21.4 months, p < 0.001)

Table 2 depicts the differences between HD and CAPD patients with regards to baseline biochemical parameters. HD patients were more likely to have lower serum albumin (28.2 ±6.7 vs 28.6 ± 6.6, p = 0.04) and serum cholesterol levels (4.1 ± 1.3 vs 4.5 ± 1.3, p = 0.001) at dialysis initiation. CAPD patients had higher haemoglobin levels than HD patients (9.1±2.3 *vs* 8.4 ± 2.1, p = 0.001) and were also on lesser doses of subcutaneous erythropoietin than HD patients [10,000 (8,000–12,000) units/week vs 12,000 (8,000–12,000) units/week, p = 0.001].

**Table 2:** Baseline biochemical parameters of patients according to dialysis modality.

Biochemical Parameter	All patients N = 340	HD patients (n = 194)	CAPD patients (n = 146)	p-value
**Serum albumin (g/L)**	28.4 ± 6.7	28.2 ±6.7	28.6 ± 6.6	0.04*
**Total Cholesterol (mmol/L)**	4.3 ± 1.4	4.1 ± 1.3	4.5 ± 1.3	0.001*
**Serum corrected calcium (mmol/L)**	2.4 ± 0.3	2.4 ± 0.3	2.4 ± 0.3	0.68
**Serum phosphate (mmol/L)**	1.4 (1.0–2.1)	1.4 (1.04–2.1)	1.5 (1.0–2.1)	0.16
**CXP (mmol**^**2**^**/L**^**2**^**)**	3.9 ± 2.3	4.0 ± 2.6	3.7 ± 1.8	0.002
**Serum haemoglobin (g/dl)**	8.7 ± 2.2	8.4 ± 2.1	9.1 ± 2.3	0.001*
**Serum Ferritin**	414.0 (204.0–714.5)	425.0 (196.0–755.0)	410.0 (206.0–677.0)	0.60
**Transferrin saturation (%)**	26.0 (15.0–44.0)	26.0 (15.0–48.1)	27.0 (16.0–41.0)	0.21
**EPO (units/week)**	10,000 (8,000–12,000)	10,000 (8,000–12,000)	12,000 (8,000–12,000)	0.001*

* p<0.05

A total of 92 deaths (27.1%) occurred over the duration of follow up. Although there were more deaths among HD patients, this was not statistically different from deaths among CAPD patients (55.4% vs 44.6% p = 0.812). There were a total of 27 CV deaths (29.3%) and 32 infection-related deaths (34.8%). A higher proportion of CV deaths was recorded among females (55.6% vs 44.4%) while a greater proportion of infection-related deaths occurred in males (53.1% vs 43.8%) [not shown in Tables].

**Fig 1:**
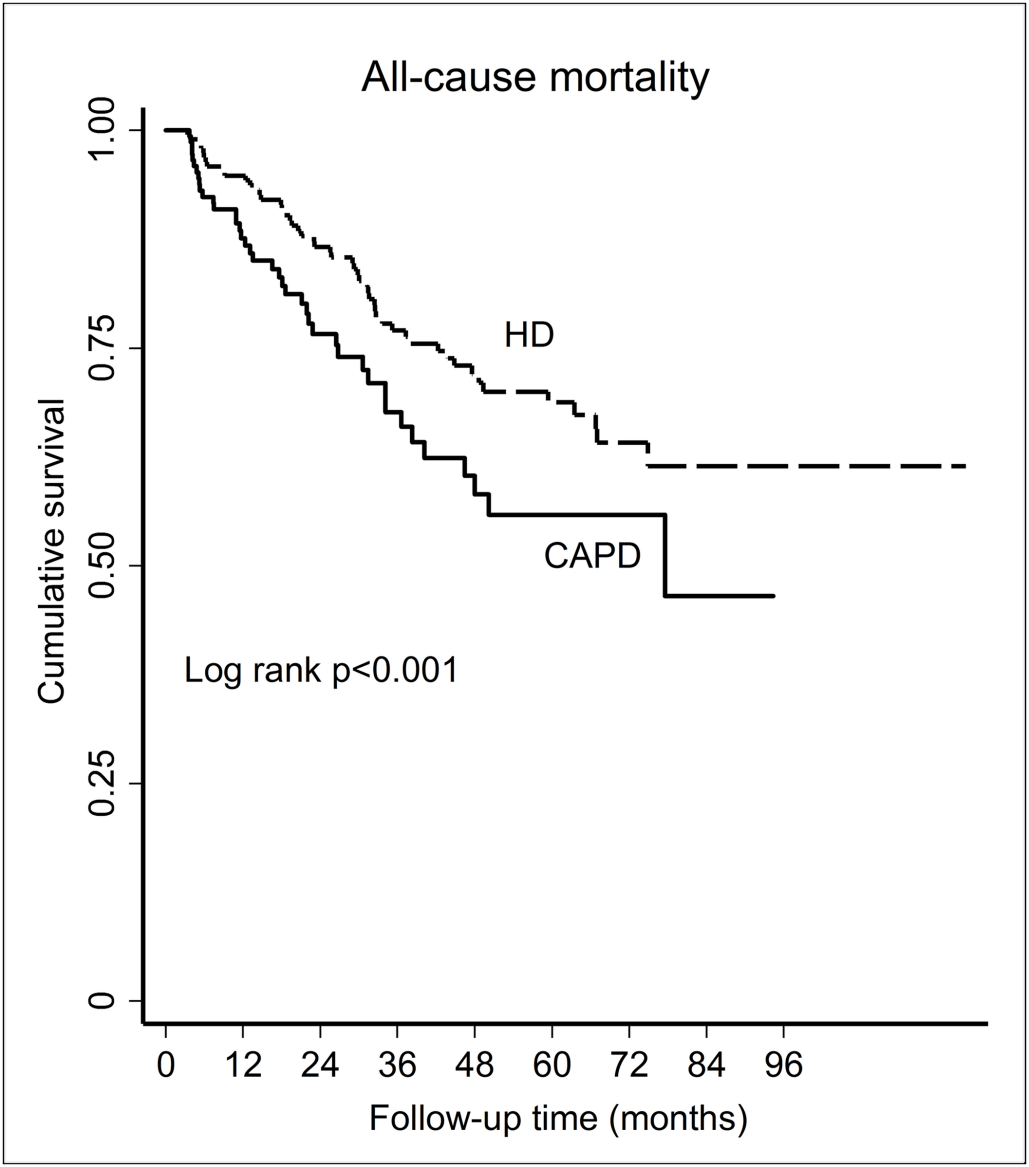
Kaplan-Meier survival curve for all-cause mortality according to dialysis modality.

**Fig 2:**
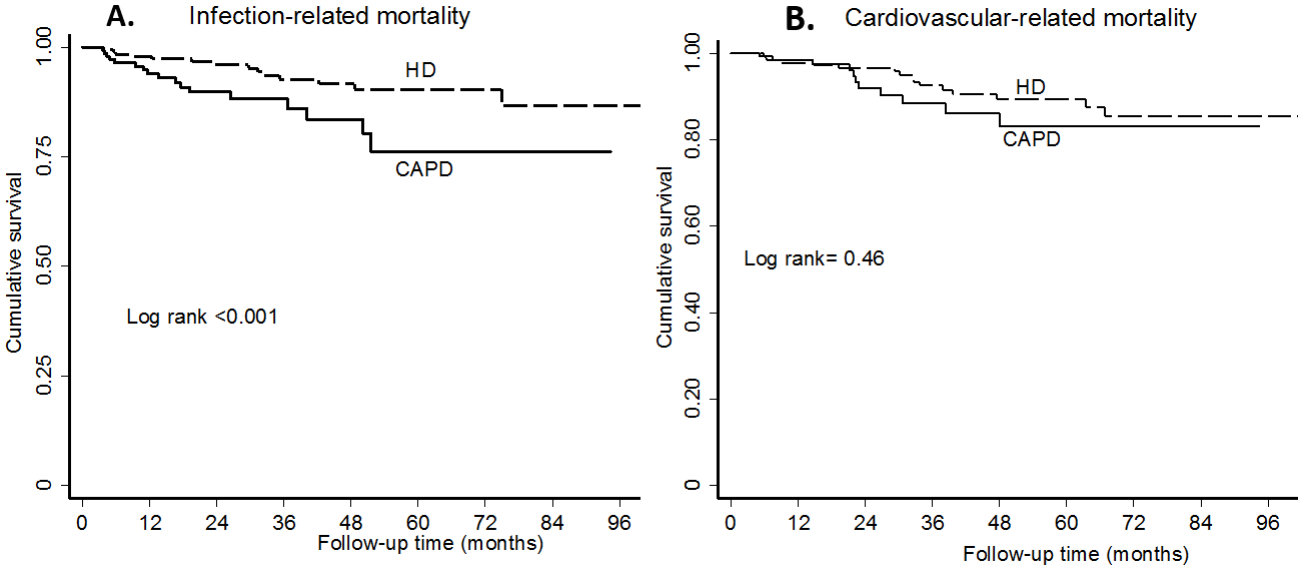
Kaplan-Meier survival curves cause-specific mortality according to dialysis modality. a. Infection-related mortality. b. Cardiovascular mortality.

Fig 1 depicts all-cause mortality survival curves of HD and CAPD patients. There was a significant difference in survival times between HD and CAPD patients (log rank test < 0.001). Approximately a third (30%) of HD and CAPD patients had died at 49.4 (CI: 47.6–63.5) months and 34.1 (CI: 30.6–36.6) months respectively. Fig 2a and 2b show the cause-specific survival curves of infection-related mortality and CV mortality respectively.

Table 3 shows the relative risks (hazard ratios) of death due to all causes and specific causes on univariate cox regression analysis. Patients on CAPD had a 62% increased risk of death from all causes as well as a greater than 2-fold increase in the risk of death from infectious causes. Significantly as well, there was a 10% reduction in the risk of death (all-cause mortality) for every unit increase in haemoglobin levels. Although it was not of statistical significance, patients with DM had almost twice the risk of death from CV causes in comparison to non-diabetics. Weight at initiation of dialysis was however significantly associated with the risk of CV death (HR: 0.97, CI: 0.95–0.99, p = 0.04).

**Table 3:** Univariate Hazard ratios for all-cause and cause-specific mortality.

Characteristic	All-cause Mortality	CV mortality	Infection-related mortality
**Modality (PD)**	1.62 (1.07–2.46)*	1.34 (0.62–2.88)	2.27 (1.13–4.60)*
**Age (years)**	1.00 (0.98–1.-02)	1.02 (0.99–1.06)	1.00 (0.97–1.03)
**Gender (male)**	0.89 (0.59–1.34)	0.83 (0.39–1.78)	1.24 (0.61–2.51)
**Distance to centre (50 Km)**	1.00 (0.99–1.00)	1.00 (0.99–1.01)	1.09 (0.88–1.35)
**Housing (Informal)**	1.28 (0.82–2.03)	1.30(0.54–3.25)	1.32 (0.61–2.85)
**Diabetes mellitus (present)**	1.43 (0.79–2.56)	1.86(0.73–4.80)	1.62 (0.62–4.23)
**Hypertension (present)**	0.88 (0.54–1.43)	0.84 (0.34–2.09)	1.24 (0.52–2.67)
**Weight (kg)**	0.99 (0.98–1.00)	0.97 (0.95–0.99)*	1.01 (0.99–1.02)
**sAlbumin (g/L)**	0.98 (0.99–1.01)	0.99 (0.94–1.06)	0.98 (0.93–1.04)
**sCholesterol (mmol/L)**	1.03 (0.86–1.23)	0.93 (0.66–1.30)	1.08 (0.68–1.72)
**Corrected Calcium (mmol/L)**	1.25 (0.61–2.57)	2.46 (0.49–12.23)	0.95 (0.25–3.63)
**CXP (mmol**^**2**^**/L**^**2**^**)**	0.86 (0.75–0.99)*	0.71 (0.55–0.90)*	0.84 (0.68–1.03)
**sHemoglobin (g/dl)**	0.90 (0.82–0.99)*	0.90 (0.75–1.07)	0.93(0.77–1.14)
**sFerritin**	1.00 (0.99–1.0007)	1.00 (0.99–1.001)	1.00 (0.99–1.00)
**EPO (per 1000units)**	0.99 (0.95–1.04)	0.99 (0.92–1.07)	0.96 (0.89–1.03)

* p < 0.05

sAlbumin—serum albumin; sCholesterol—serum cholesterol—CXP—Calcium-phosphate product.; sHaemoglobin—serum haemoglobin; sFerritin—serum ferritin; EPO—Erythropoietin

In assessing potential baseline predictors of all-cause mortality, CAPD remained an independent predictor of all-cause mortality (HR: 2.00, CI: 1.29–3.10) after adjusting for weight at dialysis commencement, comorbidity (diabetes mellitus), anaemia (using haemoglobin as a measure), and baseline serum albumin(Table 4). Likewise, baseline haemoglobin remained a predictor of all-cause mortality with a 13% reduction in risk of death from all-causes for every unit increase in haemoglobin levels (HR: 0.87 CI: 0.79–0.97, p = 0.01). Mortality risk according dialysis modality was significantly modified by diabetes mellitus status on both univariate and multivariable analyses (Table 5). Mortality risk was approximately 5 times higher among diabetics who had CAPD relative to non-diabetics on HD (HR: 4.99, CI: 2.13–11.71).

**Table 4:** Multivariate Cox regression model of the baseline predictors of all-cause mortality.

Characteristic	Hazard ratio	Confidence intervals	p-value
**Modality (PD)**	2.00	1.29–3.10	**0.002**
**Diabetes mellitus (present)**	1.67	0.92–3.02	0.09
**Hypertension (present)**	0.86	0.52–1.41	0.55
**sHemoglobin (g/dl)**	0.87	0.79–0.97	**0.01**
**sAlbumin (g/L)**	0.98	0.97–1.00	0.25
**Weight (kg)**	0.98	0.97–1.00	0.07

sAlbumin—serum albumin; sHaemoglobin—serum haemoglobin; sFerritin

**Table 5:** Univariate and Multivariate relative risks of all-cause mortality according to Diabetes status and dialysis modality.

	Unadjusted model		Adjusted Model ^#^	
	Hazard ratio	CI	Hazard ratio	CI
**DM- HD**	Ref	Ref	Ref	Ref
**DM + HD**	1.03	0.44–2.42	1.02	0.43–2.50
**DM- PD**	1.47	0.94–2.31	1.63	**1.01–2.62**∞
**DM+ PD**	**3.44**	1.54–7.67*	**4.99**	2.13–11.71*

^#^ adjusted for age, albumin, cholesterol and hemoglobin at baseline

Ref- reference group

* P < 0.001;

^∞^ p< 0.05

## Discussion

In our study, we have demonstrated that CAPD as an RRT option among predominantly rural-dwelling ESRD patients is associated with increased all-cause and infection-related risks of death. An increased risk of death from all-causes has been described in mortality outcome studies among dialysis patients in developed and developing countries. Among CAPD patients in Mexico, 1 and 3- year survival was poor at 67% and 48% respectively while Stack *et al* reported increasing adjusted relative risks for death among PD patients as duration of dialysis increased with the maximum risk demonstrable 24 months after dialysis initiation (HR 1.37, CI:1.25–1.51, p<0.001).[13, 14] However, we could not demonstrate an interaction between modality and duration on dialysis but we noticed that CAPD patients who died had a significantly short duration of follow-up than CAPD patients who remained alive (20.2 ± 16.3 *vs* 31.9 ± 22.6 months, p<0.001). The risk for all-cause mortality among CAPD patients increased after adjustment for DM, systemic hypertension, and select baseline parameters (HR 1.98, CI: 1.28–3.05, p = 0.002). The modifying effect of DM (as a cause of ESRD and comorbidity) on mortality in PD patients is well known.[15] Vonesh *et al* reported an age and co-morbidity adjusted average mortality risk ratio of 1.22 among United States (US) Medicare incident dialysis patients with DM.[16] There was a significant increase in the risk of death among our DM patients on PD (adjusted HR: 4.99, CI: 2.13–11.71, p<0.001) [Table 5]. Differences in patient characteristics at baseline may explain our higher mortality rates. Patients in PKDC were at baseline more malnourished than the US Medicare group with lower mean serum albumin levels. Nineteen percent of patients (19.0%) within the US cohort on PD had serum albumin level <31g/L in comparison to 56.9% of our patients. Serum albumin is a recognised marker of malnutrition in dialysis patients, and in turn, malnutrition a known predictor of poor outcome in PD. Baseline haemoglobin levels were also lower among our patients at dialysis initiation.

Over the decades, infection-related deaths have accounted for the majority of non-CV deaths among dialysis patients.[17, 18] The risk of death from infection-related causes was significantly higher among our PD patients with peritonitis being the commonest cause of infection. The relationship between peritonitis and outcomes in PD is well-established. An analysis of the Australian-New Zealand data base demonstrated the notable role peritonitis played in the mortality of PD patients.[19] Infection rates vary among peritoneal dialysis modalities with the lowest risk associated with automated peritoneal dialysis (APD) systems.[20] In our cohort, CAPD was the PD modality utilized and may have accounted for the observed peritonitis and death occurrences. Peritonitis rates in PD programs are usually a reflection of the standard of care in PD programs. Factors that can be attributed to the possibility of sub-optimal standard of care among our patients are technical manpower shortages and lack of adequate infrastructure in health services systems in rural settings. In rural, underdeveloped regions of china where there are significantly less doctors and trained health staff in PD programs, PD outcomes have been noted to be poor in comparison to PD patients with access to adequately staffed urban units.[21, 22] The relative lack of nearby standard PD services in our patients’ locale is brought to bear in the average distance travelled to access care (114.3 ± 70.2 km). Cost considerations in making these journeys will most certainly deter early presentation for prompt intervention when peritonitis symptoms develop. Our patients are largely unemployed and those accepted unto the chronic dialysis program are given 1200 Rands (approximately $85) per month in form of a social grant by the provincial government. The lack of an association between infection-related mortality and type of housing mirrors the positive impact of good housing conditions in infection control. A significantly higher proportion of PD patients dwelt in formal houses which are characterized in South Africa by the presence of running water and proper sewage disposal systems. In an attempt not to make invalid associations, we did not further assess the relationship among risk of infection-related death, peritonitis and type of housing because of the small numbers.

Cardiovascular causes account for the majority of deaths among chronic dialysis patients.[23] In our cohort of patients however, deaths related to CV causes accounted for only 29.3% of all deaths with body weight at dialysis initiation being the only significantly associated factor for CV mortality. We recognise that this low proportion of CV deaths can be accounted for by selection bias whereby patients who are healthier, younger, and with less comorbidities and CV disease burden are dialysed. This is apparent in the mean age of our patients (36.1 ± 11.9 years), mean BMI (23.9 ± 5.5 Kg/m^2^) and the percentage of patients with DM (10.3%) and hypertension (25.9%) in a predominantly black population of patients. Due to the current dialysis-rationing policy operational in government-funded dialysis centres in South Africa (ours inclusive), stringent criteria are applied in accepting ESRD patients to the maintenance dialysis program.[24] The exclusion criteria under this policy are factors that are known to be associated with poor CV outcomes. As such patients who are > 60 years, diabetic (if > 50 years), morbidly obese (>BMI.35kg/m2) and those with advanced and irreversibly progressive cardiac, CV or peripheral vascular disease are not accepted on to the program.

Peritoneal dialysis has the potential of being a preferred RRT option in developing countries as it could serve rural dwelling patients who commonly live far away from in-centre HD units which are usually cited in urban areas. However, poorer patient outcomes in PD patients may prevent its optimal usage among this group of patients in a country like South Africa. Even though this study has described poorer survival among PD patients, this should not discourage the use of PD as an RRT modality given that it can be relatively cheaper than HD, requiring minimal supervision by trained nephrologists and creating time for the patient to be otherwise gainfully employed.[25] The opportunity to be able to keep a job is especially important in a young ESRD population like ours. Moreover, we have previously reported comparable outcomes for patients on PD in South Africa with those from developed countries.[26]

Our study is not without a number of limitations. Its retrospective design (with the inherent problem of missing records) made it difficult to assess all relevant socioeconomic, clinical and laboratory parameters known to be associated with mortality in dialysis patients as these had not been adequately documented. From the foregoing also, the efficiency of the delivered dose of therapy (HD and PD) over the duration could not be reliably extracted. However given the socioeconomic and demographic landscape of South Africa, this type of study can be prospectively set up to investigate mortality outcomes among rural dwellers. Similarly, comparative prospective studies assessing the outcomes between rural and urban dwellers will further define the clinical epidemiology of dialysis therapies in South Africa.

In conclusion, we have established that in rural dwelling ESRD patients receiving dialysis therapies in South Africa, CAPD is associated an increased risk of all-cause and infection-related mortality. We believe that poor access to health care facilities plays a contributory role in infection-related mortality and we thus advocate for the establishment of CAPD centres in rural areas of South Africa.
